# Geographical Classification of Saffron (*Crocus Sativus* L.) Using Total and Synchronous Fluorescence Combined with Chemometric Approaches

**DOI:** 10.3390/foods12091747

**Published:** 2023-04-23

**Authors:** Ouarda El Hani, Juan José García-Guzmán, José María Palacios-Santander, Khalid Digua, Aziz Amine, Said Gharby, Laura Cubillana-Aguilera

**Affiliations:** 1 Laboratory of Process Engineering and Environment, Faculty of Sciences and Techniques, Hassan II University of Casablanca, P.A. 149, Mohammedia 28810, Morocco; ouardaelhani1997@gmail.com (O.E.H.);; 2 Department of Analytical Chemistry, Institute of Research on Electron Microscopy and Materials (IMEYMAT), Faculty of Sciences, Campus de Excelencia Internacional del Mar (CEIMAR), University of Cadiz, Campus Universitario de Puerto Real, Polígono del Río San Pedro S/N, 11510 Puerto Real, Cádiz, Spain; juanjo.garciaguzman@uca.es (J.J.G.-G.);; 3 Biotechnology Analytical Sciences and Quality Control Team, Laboratory of Analysis Modeling, Engineering, Natural Substances and Environment, Polydisciplinary Faculty of Taroudant, University Ibn Zohr, Agadir 80000, Morocco

**Keywords:** saffron, synchronous fluorescence, emission-excitation matrix, geographical classification, principal component analysis, linear discriminant analysis

## Abstract

There is an increasing interest in food science for high-quality natural products with a distinct geographical origin, such as saffron. In this work, the excitation-emission matrix (EEM) and synchronous fluorescence were used for the first time to geographically discriminate between Moroccan saffron from Taroudant, Ouarzazate, and Azilal. Moreover, to differentiate between Afghan, Iranian, and Moroccan saffron, a unique fingerprint was assigned to each sample by visualizing the EEM physiognomy. Moreover, principal component analysis (LDA) and linear discriminant analysis (LDA) were successfully applied to classify the synchronous spectra of samples. High fluorescence intensities were registered for Ouarzazate and Taroudant saffron. Yet, the Azilal saffron was distinguished by its low intensities. Furthermore, Moroccan, Afghan, and Iranian saffron were correctly assigned to their origins using PCA and LDA for different offsets (Δλ) (20–250 nm) such that the difference in the fluorescence composition of the three countries’ saffron was registered in the following excitation/emission ranges: 250–325 nm/300–480 nm and 360–425 nm/500–550 nm. These regions are characterized by the high polyphenolic content of Moroccan saffron and the important composition of Afghan saffron, including vitamins and terpenoids. However, weak intensities of these compounds were found in Iranian saffron. Furthermore, a substantial explained variance (97–100% for PC_1_ and PC_2_) and an important classification rate (70–90%) were achieved. Thus, the non-destructive applied methodology of discrimination was rapid, straightforward, reliable, and accurate.

## 1. Introduction

Biomedical research has highly recommended the use of herbal medicines instead of synthetic and chemical products because of their safety profile and potential effectiveness [1]. Saffron (stigma of *Crocus Sativus* L.), which belongs to the family of *Iridaceae* flowering plants, is the highest-priced spice in the world owing to its laborious harvesting procedures and pharmaceutical benefits. These include antianxiety, anti-inflammatory, antidepressant, anticancer, antitussive, anti-Alzheimer’s, and anticonvulsive effects [2,3,4]. 

Among the worldwide saffron production countries are, obviously, Morocco, Afghanistan, and Iran [5]. The disparity in the agronomic and climatic conditions in saffron cultivation areas has always revealed differences in the chemical composition of this plant. Thus, the price of saffron fluctuates [6,7]. For this reason, identification of the clear geographical origin of saffron is extremely important [8,9]. From this point, the development of analytical methods for saffron’s geographical authenticity determination is highly recommended [10,11]. Thus, the most frequently applied methods are chromatography [12], attenuated total reflectance–Fourier transform infrared spectroscopy (ATR-FTIR) [13,14], and inductively coupled plasma atomic emission spectroscopy/mass spectroscopy (ICP-AES/ICP-MS) [15,16]. Despite the authenticity discrimination achieved by these methods, they necessitate long sample preparation steps and require qualified personnel [17]. There is currently a considerable demand for analytical methods that are easy to use, quick, accurate, sensitive, and relatively cheap; require only small amounts of reagents; and are non-destructive. Indeed, UV–vis and fluorescence spectroscopy are the cases [18,19,20]. The spectrophotometric measurement of saffron was often used to evaluate the saffron’s bitterness, flavor, aroma, and color pigmentation [20]. Furthermore, fluorescence has become increasingly popular in a wide range of disciplines, including herbal medicines [21,22], cosmetics [23], and environmental assessment [24]. Unfortunately, conventional fluorescence is not recommended for the analysis of the natural matrix products such as saffron because of the presence of multiple fluorescence compounds [25]. Consequently, the application of total fluorescence or excitation-emission matrix (EEM) enables the successive measurement of the conventional fluorescence spectra by changing the excitation wavelength to improve the signal resolution and selectivity [26,27]. Along with this, synchronous fluorescence spectroscopy (SFS) is based on an important factor called the offset, which is the difference between the excitation and emission wavelengths. For good food authentication, many offsets should be investigated. This fluorescence mode has revealed many merits, including a simple spectrogram, high selectivity, and decreasing scattering of light interference [28,29,30]. 

Certainly, saffron contains different compounds [31,32] including polyphenols (phenolic acids, tannins, stilbenes, lignin, phytosterols, amino acids, and flavonoids) [33,34,35,36], quinones [37], vitamins [38,39], and terpenoids [40,41,42]. These constituents are among the well-known intrinsic fluorescent compounds that occur in plants, and they are consequently appropriate for fluorescence analysis [43,44,45]. To our knowledge, the geographical discrimination of saffron based on fluorescence spectroscopy was investigated for the first time in this work. Chemometric approaches were implemented for food authentication to enable the extraction of the relevant information by evaluating the multi-dimensional fluorescence dataset to find the feature pattern or “fingerprint” relating to the geographical authenticity of samples from different areas. Principal component analysis (PCA) and linear discriminant analysis (LDA) remain the most widely known multivariate analysis methods in the field of food geographical discrimination for the exploitation and classification of the acquired dataset [46,47,48]. 

In consideration of the aforementioned, this work aims to develop a straightforward, rapid, and accurate method for tracing the provenance of saffron samples from different regions in Morocco and also to discriminate between Afghan, Iranian, and Moroccan saffron using EEM results and the SFS dataset of different offsets combined with PCA and LDA approaches.

## 2. Materials and Methods

### 2.1. Chemicals and Saffron Samples

Methanol 99.9% and formic acid were purchased from PANREAC (Castellar del Vallès, Spain). Distilled water (18 MΩ cm) was obtained from a Millipore Milli-Q system (Bedford, MA, USA).

A set of saffron samples from three different countries was selected for the study. Moroccan saffron stigmas are cultivated in the southwestern provinces (Taroudant, Ouarzazate, and Azilal) [49,50]. Table 1 summarizes the well-known production areas in Morocco with their geographical coordinates. Eighteen different Moroccan saffron samples (at least two samples from each indicated location) were directly acquired from producers or cooperatives to guarantee their geographical origin, traceability, and genuineness (samples were collected in 2021 and 2022). Moreover, well-defined samples were supplied from Afghanistan (10 samples) and Iran (10 samples), which were purchased from commercial redistributors. The samples were kept in the absence of light until their analysis. Detailed information about the non-Moroccan samples was unavailable; only the country of origin was reported. 

### 2.2. Saffron Extracts Preparation 

Before fluorescence measurements, saffron stigmas were treated following the extraction protocol: 25–400 mg of Crocus stigmas were weighed and placed in 20 mL of the extracting solvent, H_2_O (100%), methanol (100%), H_2_O:methanol (1:1), and H_2_O:methanol:formic acid (FA) (50:49:1). The previous solution was stirred using a SONOPULS HD2200 Ultrasonic Homogenizer (BANDELIN electronic GmbH & Co., KG, Berlin, Germany) with an amplitude of 20%. After 5–40 min of the extraction, the resultant mixture was centrifuged during 2 min at 10,000 rpm and filtered through a 0.45 μm nylon syringe filter. The prepared extract was diluted ten times using methanol (1/10) and further measured with total and synchronous fluorescence spectroscopy.

### 2.3. UV–Vis Spectrophotometric Analyses of the Aqueous Extracts 

The UV–vis saffron aqueous extracts were prepared according to the standard method ISO 3632-1:2003 used for saffron spectroscopic measurements detailed in the following work [51,52]. The involved method is well known for the detection of three main bioactive constituents of saffron samples (the three major sensory attributes that contribute to saffron quality discrimination): picrocrocin (responsible for the flavor/bitterness), safranal (which gives the aroma characteristics), and crocin (which gives the saffron color). The specific peaks of the previous compounds appeared at the following wavelengths: 253, 331, and 443 nm, respectively. 

The spectrophotometric measurements were recorded using a JENWAY UV–vis spectrophotometer (UK), model 6850 UV–vis double beam with a 1 cm matched cell. The spectra were acquired in the range of 200–700 nm against the corresponding blank reagents. For each saffron sample, three replicates were investigated.

### 2.4. EEM and SFS Data Acquisition

Fluorescence spectra were acquired on a FP-6500 spectrofluorometer (Jasco, Spain). During the analysis, saffron extracts were filled into a 10 mm quartz cell, and the measurements were recorded at room temperature. The spectrum of each sample was collected in triplicate. 

For both total and synchronous fluorescence modes, excitation and emission monochromator slit widths were both fixed at 5 nm and the scan rate at 500 nm min^−1^. 

EEM contour maps were acquired in the excitation and emission scanning intervals of 250–500 nm and 300–600 nm, respectively. Thus, a set of successive emission spectra were registered for each measured sample to produce a three-dimensional matrix (λ_ex_, λ_em_, fluorescence intensity). EEM maps were obtained by plotting the fluorescence intensities as a combined function of the excitation and emission wavelengths. Rayleigh and Raman scattering were roughly corrected by subtracting the EEM matrix of the blank solution.

For synchronous measurements, different wavelength offsets (Δλ = λ_ex_ − λ_em_) were investigated, including Δλ_1_ = 20 nm (280 nm < λ_em_ < 700 nm), Δλ_2_ = 40 nm (280 nm < λ_em_ < 700 nm), Δλ_3_ = 60 nm (280 nm < λ_em_ < 690 nm), Δλ_4_ = 120 nm (320 nm < λ_em_ < 700 nm), Δλ_5_ = 150 nm (350 nm < λ_em_ < 700 nm), Δλ_6_ = 200 nm (400 nm < λ_em_ < 700 nm), and Δλ_7_ = 250 nm (450 nm < λ_em_ < 600 nm).

The spectrofluorometer was interfaced with a computer supplied with Fluorimetro software ver. 1.55.00 (Thermo Scientific, Waltham, MA, USA) for spectral data acquisition. 

### 2.5. Chemometric Analyses of SFS Datasets

The acquired SFS datasets generally showed multiple peaks with variable intensities. Therefore, the geographical discrimination could not be visually identified, especially when many samples were investigated. Hence, the application of multivariate analysis is of vital importance. Indeed, PCA and LDA were performed to analyze the SFS results using JMP software (Pro 14.0.0). 

For each Δλ, the PCA approach was used as an unsupervised exploratory statistical procedure to visualize the acquired fluorescence dataset and also to identify any possible clustering of saffron samples according to their geographical authenticity. PCA was based on the reduction of the data dimension and transforming the original variables (the whole range of wavelengths) to synthetic non-correlated variables called principal components (PCs), retaining as much information as possible. The choice of the number of PCs is generally related to their explained variance [53]. PCA scores for each wavelength offset can be constructed into two dimensions with just PC_1_ and PC_2_ or with the first three dimensions from PC_1_, PC_2_, and PC_3_ depending on the number of PCs that have been pre-defined. The score plot enabled the visualization of samples distribution in the PCA graphical display.

LDA was employed to determine the linear canonical discrimination function, retaining the maximized ratio of between-class and within-class variance [54]. LDA required several samples superior to the number of variables (wavelengths). For this reason, specific emission wavelengths that corresponded to the most discriminating peaks for the saffron samples were taken into consideration. Thus, the characteristic peaks that were fixed as inputs in LDA analysis for each offset are detailed as follows: 301, 516, 551, and 590 nm (for offset Δλ_1_); 320, 534, 563, and 630 nm (for offset Δλ_2_); 288, 342, 560, 581, and 630 nm (for offset Δλ_3_); 352, 442, 470, 525, and 575 nm (for offset Δλ_4_); 370, 442, 481, and 596 nm (for offset Δλ_5_); 420, 450, and 502 nm (for offset Δλ_6_); and 480, 511, and 540 nm (for offset Δλ_7_). 

## 3. Results and Discussion 

### 3.1. Spectrophotometric Measurements of Saffron Aqueous Extracts

The saffron’s colorant, aromatic, and flavoring properties, which were due to the presence of the main secondary compounds including crocin, safranal, and picrocrocin, respectively, were evaluated by spectrophotometry in the range of 200–700 nm. The UV–vis spectra of Moroccan (coming from Taroudante, Ouarzazate, and Azilal), Afghan, and Iranian saffron extracts are illustrated in Figure S1. It can be clearly observed that all the UV–vis spectra show three well-defined absorption bands at 253, 331, and 443 nm. Moroccan saffron coming from Taroudant and Ouarzazate presented the maximum absorbance values. After that, the Azilal and Afghan saffron samples showed lower absorbances. Next, Iranian saffron offered the lowest absorbance values. 

Nevertheless, it was challenging to discriminate between Moroccan (Azilal) and Afghan saffron samples since most of both samples’ UV–vis spectra were overlapped in the picrocrocin peak around 443 nm (as shown in Figure S1). Hence, spectrophotometry gave just partial information about the saffron quality. For this reason, applying another analytical method (such as fluorescence) was very necessary to distinguish between the investigated saffron samples.

### 3.2. Optimization of the Saffron Extracts Preparation for Fluorescence Analysis

#### 3.2.1. Optimization of the Extraction Solvent 

For the selection of the extraction solvent, four solvents including H_2_O (100%), methanol (100%), H_2_O:methanol (1:1), and H_2_O:methanol:FA (50:49:1) were evaluated for the extraction of 500 mg of the Moroccan saffron sample (Taliouine) during 30 min. Following the extraction protocol detailed in Section 2.2, the prepared extracts were measured by EEM fluorescence, as shown in Figure S2. The lowest fluorescence intensities were measured when using H_2_O and methanol in the proportion of 100%. Moreover, medium intensities were noticed for H_2_O:methanol (1:1) EEM. Moreover, the highest fluorescence intensities were displayed for H_2_O:methanol:FA (50:49:1) EEM. Thus, the last solvent was selected as the adequate extraction solvent for further experiments.

#### 3.2.2. Optimization of the Saffron Weight

Upon establishing the adequate extraction solvent, four different saffron weights were assessed; namely, 25, 100, 200, and 400 mg were extracted in H_2_O:methanol:FA (50:49:1) for 30 min. The prepared extracts were measured for EEM fluorescence. Figure S3 illustrated the EEM contour of each evaluated weight. Based on the results, the fluorescence proportionally increased from 25 to 200 mg. After that, intensities remained constant. Hence, 200 mg was chosen for further experiments.

#### 3.2.3. Optimization of the Extraction Time 

After determining the adequate extraction solvent and fixing the saffron weight, various extraction times were tested, including 5, 10, 20, and 40 min. EEMs maps corresponding to each evaluated time are shown in Figure S4. Fluorescence intensities significantly increased from 5 to 20 min, and then, the intensities reached the equilibrium state. For this reason, 20 min was fixed as the adequate extraction time for further experiments.

### 3.3. Fingerprinting of Saffron Extracts Using Emission-Excitation Matrix (EEM)

**Figure 1:**
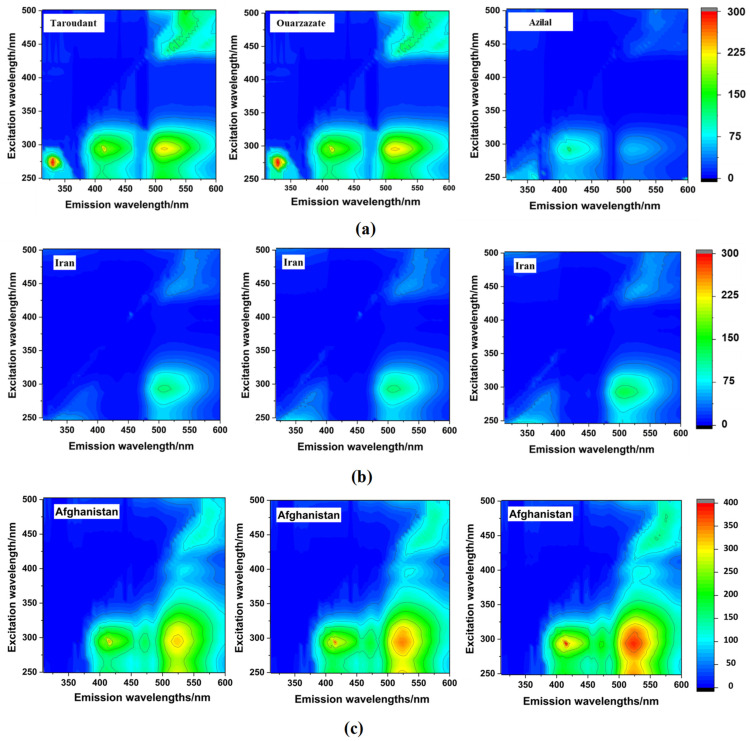
EEMs maps of Moroccan (**a**), Iranian (**b**), and Afghan (**c**) saffron samples.

Figure 1 shows the contour maps of the investigated saffron samples from Morocco (detailed EEMs of all Moroccan samples are presented in Table S1), Afghanistan, and Iran. These maps were obtained using the instrumental parameters indicated in Section 2.4. The variation of the coloration corresponds to the variations of the fluorescence intensities, from cooler shading in blue (weak intensities) to warmer shading in red (strong intensities). Moreover, the contours were obtained by linking the points of equal fluorescence intensity. As can be seen in all EEMs, a clear difference can be observed between saffron samples from Morocco (national discrimination) and saffron samples from Afghanistan and Iran (international discrimination) by the visual evaluation of the obtained EEMs physiognomy. 

In Moroccan EEM maps, four fluorescence regions were obviously distinguished. Each region corresponded to specific excitation/emission ranges pairs: region 1 (R1): Ex (250–325 nm)/Em (300–375 nm); region 2 (R2): Ex (250–325 nm)/Em (380–455 nm); region 3 (R3): Ex (250–375 nm)/Em (480–600 nm); and region 4 (R4): Ex (425–500 nm)/Em (480–600 nm). The difference between these samples was defined in terms of the fluorescence intensities. The lower fluorescence intensities were registered for the Azilal saffron samples. The high fluorescence intensities characterized the saffron from Taroudant and Ouarzazate. Therefore, clear differences in the EEMs physiognomy were observed between Moroccan saffron samples according to their regional origin. 

Fluorescence mapping of Afghan saffron was characterized by the presence of three out of four characteristic fluorescence regions found in Moroccan EEMs (R2, R3, and R4) and by the absence of R1, which probably corresponded to some phenolic compounds and amino acids present in saffron, such as syringic acid and tryptophan, respectively, both considered in the literature as the main fluorophores found in food [51,52]. Furthermore, Afghan EEMs were characterized by the apparition of two other regions, namely region 5 (R5): Ex (250–325 nm)/Em (455–480 nm) and region 6 (R6): Ex (360–425 nm)/Em (500–550 nm), which are supposedly attributed to the presence of some vitamins such as vitamin A and vitamin B2 and terpenoids such as saponins and crocusatins, which are owing to the fluorescence features of these compounds in the indicated ranges [53,54]. Moreover, Iranian EEMs presented similar Moroccan EEM fluorescence regions with the absence of R2 and lower intensities in other registered regions. R2 characterizes the fluorescence phenomenon of some polyphenols including sinapic and gentisic acids [51,55]. All these facts confirm the significant difference in terms of the fluorescence composition of Moroccan, Iranian, and Afghan saffron samples. Accordingly, the EEM of each country is unique, thus reflecting the saffron’s molecular fingerprint. However, one of the EEM limitations is the overlapping of near fluorophores spectra. Thus, to enhance the resolution, another fluorescence mode measurement, synchronous fluorescence, was employed.

### 3.4. Synchronous Fluorescence Spectra (SFS) of Saffron Extracts

**Figure 2:**
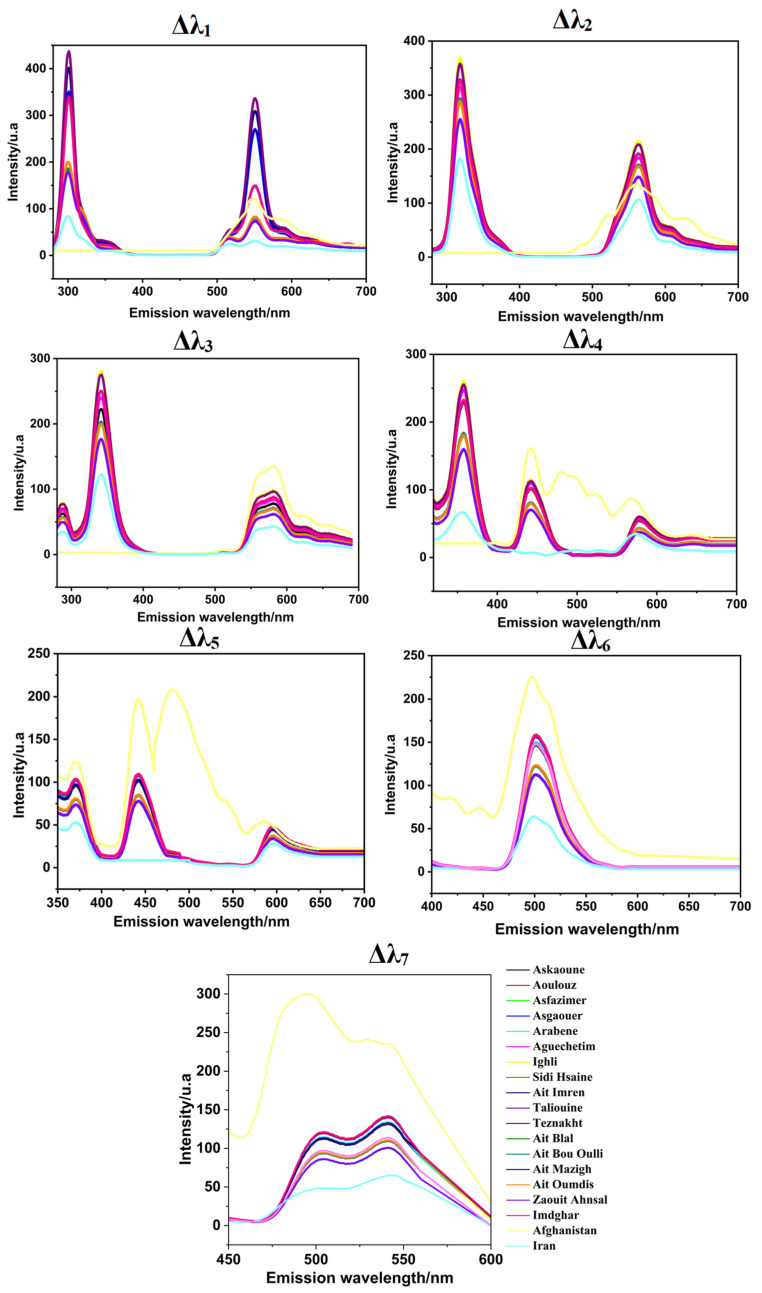
SFS results at different offsets (Δλ_i_, with *i* = 1 to 7) for Moroccan (Taroudant, Ouarzazate, and Azilal), Afghan, and Iranian saffron samples.

For each saffron sample, the synchronous dataset was collected by simultaneous scanning of the excitation and the emission with a fixed difference (Δλ_i_, with *i* = 1 to 7). The SF spectra showed relevant resolution, as shown in Figure 2. 

### 3.5. Geographical Origin Discrimination of Saffron Samples

#### 3.5.1. Principal Component Analysis (PCA) of the Synchronous Saffron Samples Spectra

**Figure 3:**
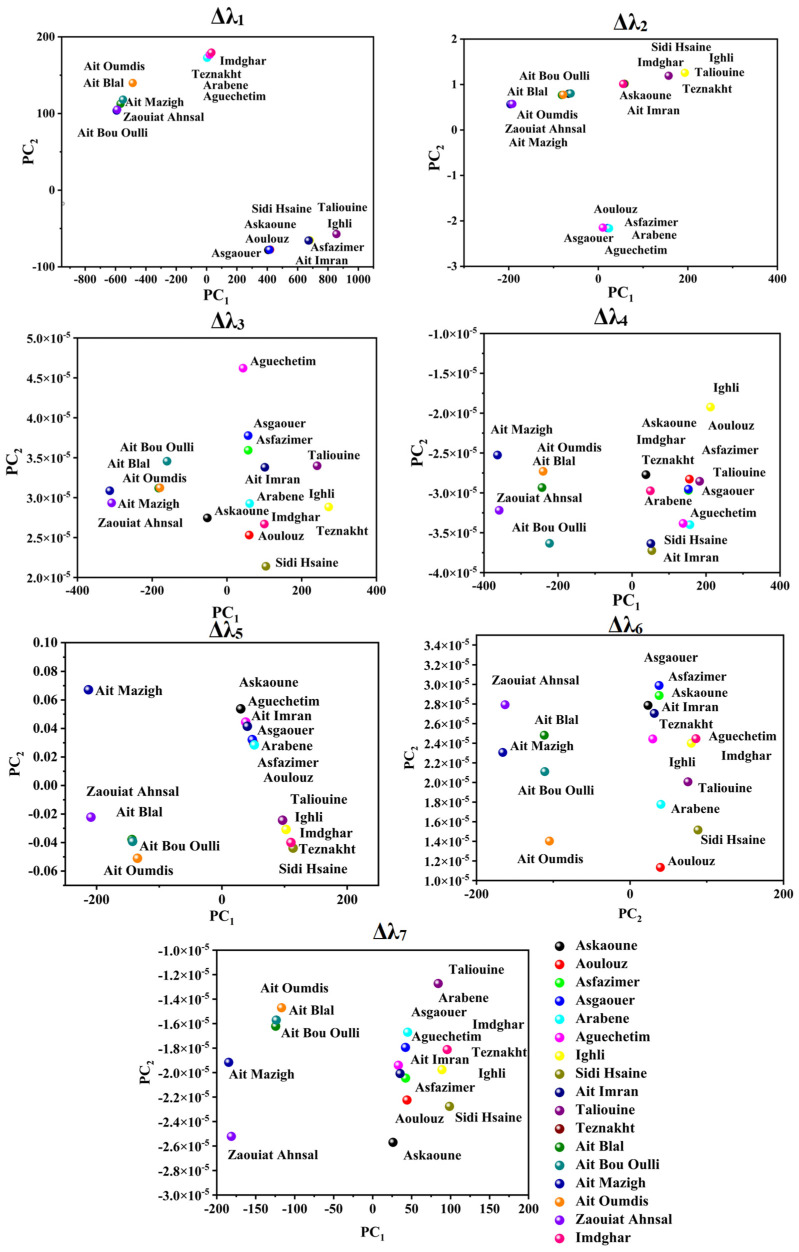
Score plots of Moroccan saffron samples according to the PC_1_ and PC_2_ using different offsets (Δλ_i_, with *i* = 1 to 7). Askaoune, Aoulouz, Asfazimer, Asgaouer, Arabene, Aguechetim, Ighli, Sidi Hsaine, Ait Imran, and Taliouine from Taroudant; Imdghar and Tazenakht from Ouarzazate; Ait Mazigh, Zaouiat Ahansal, Ait Bou Oulli, Ait Blal, Ait Oumdis, and Ait Tamlil belonged to the Azilal Province.

**Figure 4:**
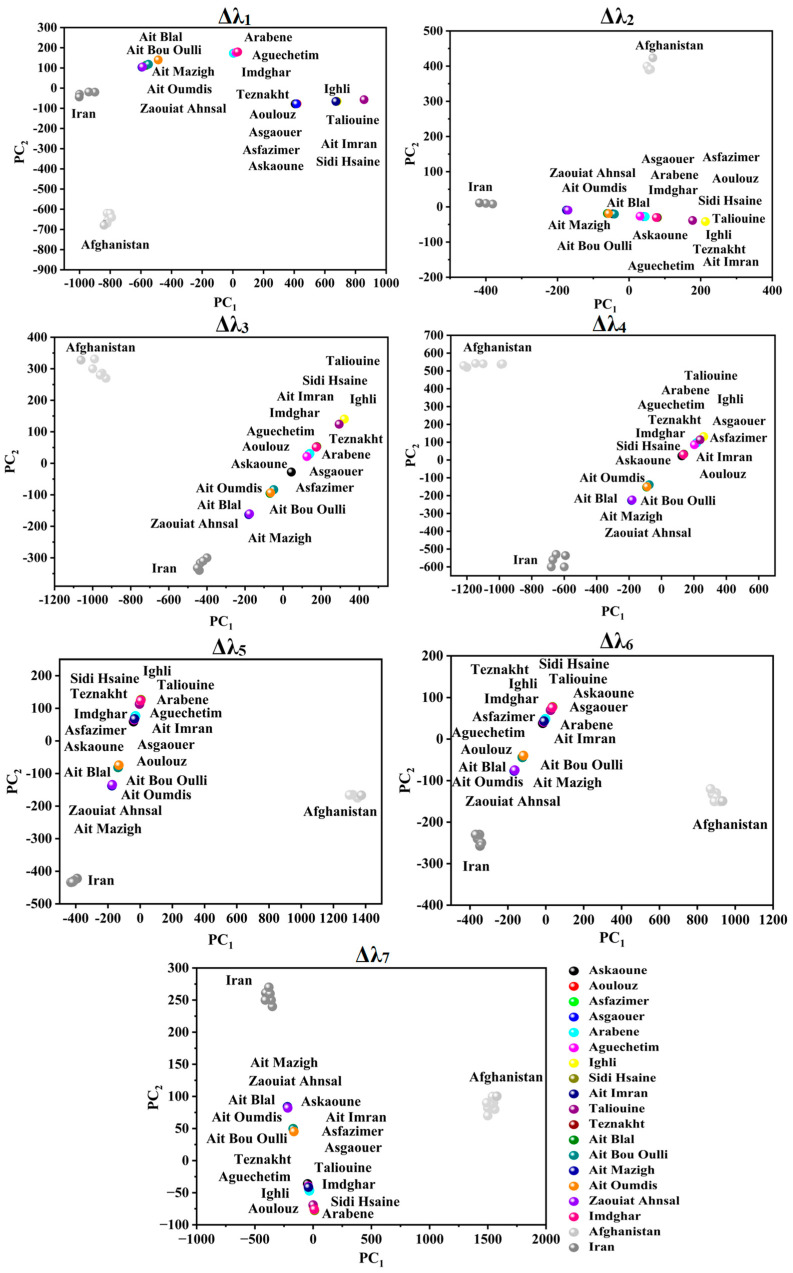
Score plots of Moroccan, Afghan, and Iranian saffron samples according to PC_1_ and PC_2_ using different offsets (Δλ_i_, with *i* = 1 to 7).

PCA was applied to attempt the visualization of the acquired SFS and the identification of any possible grouping aspects of the different investigated saffron samples using the seven studied offsets. Figure 3 and Figure 4 illustrate the projection of the samples in PCA space. The selected number of PCs in all offset assessments was two PCs due to the significant variance explained by these two dimensions, as summarized in Table 2. The explained calibration variance ((var)_c_) was from 98% to 99.2% for Moroccan saffron distribution, and the explained calibration variance varied between 97% and 100% for Moroccan, Afghan, and Iranian saffron samples. Moreover, PCA models were validated for the saffron geographical origin using the cross-validation method with six cancelation samples (two samples from each province for Moroccan saffron discrimination and two samples from each country for the international classification). The total explained validation variance ((var)_v_) for the first two PCs ranged from 80% to 99% for Moroccan saffron sample discrimination as well as for Afghan, Iranian, and Moroccan saffron sample classification, indicating the excellent performance and accuracy of the PCA models.

Successful origin authenticity of Moroccan saffron was obtained especially when using Δλ_4,5,6,7_ (as shown in Figure 3) in such a manner that Azilal saffron samples, which were represented in Ait Mazigh, Ait Blal, Ait Bou Oulli, Zaouiat Ahansl, and Ait Oumdis (distributed in the negative side of PC_1_), were separated from the samples from Taroudant and Ouarzazate (distributed in the positive side of the PC_1_ axe) and represented by the following samples: Askaoune, Aoulouz, Asfazimer, Asgaouer, Arabene, Aguechetim, Ighli, Sidi Hsaine, Ait Imran, Taliouine, Imdghar, and Teznakht. In addition, saffron samples of Tarosudante and Ouarzazate are characterized by similar distribution in PCA space. Certainly, the achieved geographical grouping of the investigated samples is due to the difference in fluorescence intensities between Azilal saffron SF spectra (weak emission intensities) and Taroudante and Ouarzazate saffron (strong emission intensities), which can be explained by the disparity of the climate circumstances and soil properties in these areas. Similarly, Ouarzazate and Taroudant are geographically neighboring, and they were deemed to be vital saffron production areas in Morocco because of the suitable conditions of saffron cultivation, such as the important soil- and runoff water-mineral content of Zn, Fe, Mn, Mg, and Cu and semiarid bio-climatic range (a little rainfall throughout the year, ranging from 140 and 214 mm) [15,49,55]. 

Concerning Moroccan, Afghan, and Iranian samples’ PCA scores (as illustrated in Figure 4), a fingerprint of saffron distribution can be significantly attributed to the place of origin.

For Δλ_1_ and Δλ_2_, noteworthy discrimination of Afghan saffron from the remaining samples was obtained based on PC_2_ due to the absence of emission at 280–350 nm (for Δλ_1_) and 300–400 nm (for Δλ_2_) in the Afghan SF spectra. Moreover, Iranian saffron was differentiated from Afghan and Moroccan saffron (especially Taroudant and Ouarzazate).

Considering Δλ_3_ and Δλ_4_, PC_1_ noticeably separated Afghan saffron. Moreover, PC_2_ separated Iranian (negative side) from Afghan, Taroudant, and Ouarzazate saffron (positive side), while Azilal saffron was not well classified. 

According to Δλ_5_ and Δλ_6_, PC_1_ divided samples into three classes: Afghan saffron on the positive side (high emission intensities), Moroccan saffron in the middle, and Iranian saffron on the negative side (low emission intensities), whereas Moroccan saffron was distinguished from the other samples according to PC_2_. 

Regarding Δλ_7_, PC_1_ separated the Afghan saffron in its positive side from the remaining samples.

#### 3.5.2. Linear Discriminant Analysis (LDA) of the Synchronous Saffron Samples’ Spectra 

**Figure 5:**
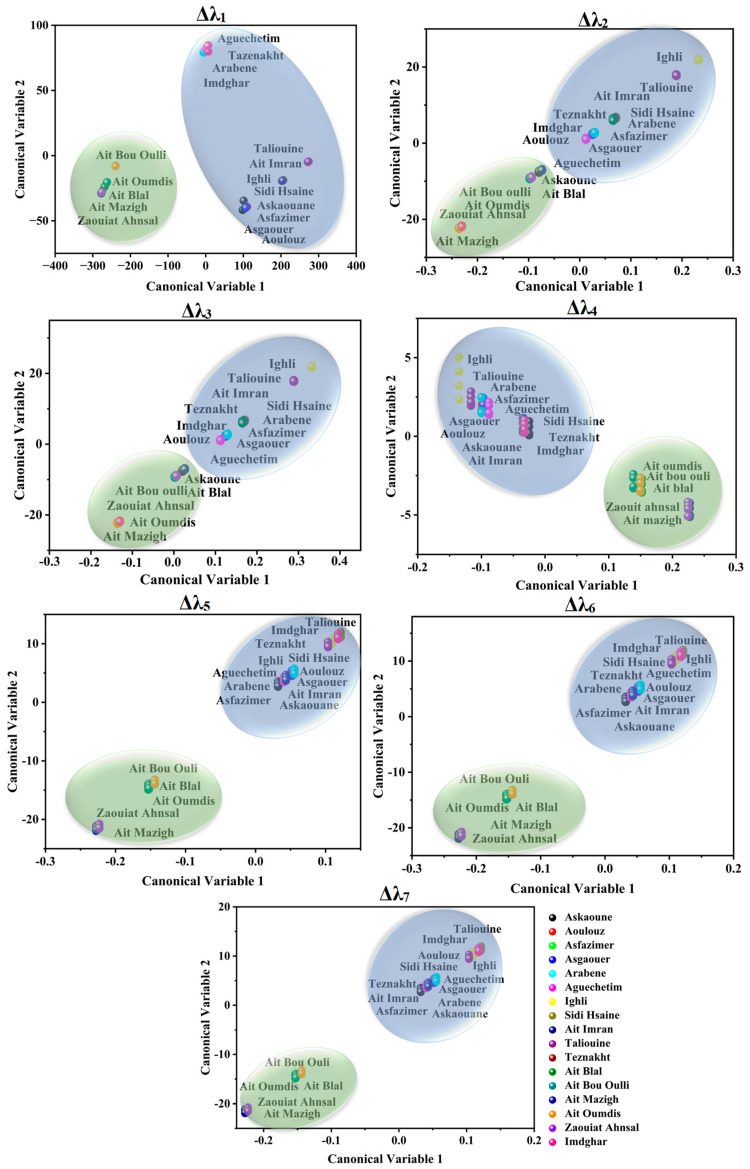
LDA scores applied on Moroccan saffron SFS spectra using different offsets (Δλ_i_, with *i* = 1 to 7). Askaoune, Aoulouz, Asfazimer, Asgaouer, Arabene, Aguechetim, Ighli, Sidi Hsaine, Ait Imran, and Taliouine from Taroudant (Blue surface); Imdghar and Tazenakht from Ouarzazate (Blue surface); Ait Mazigh, Zaouiat Ahansal, Ait Bou Oulli, Ait Blal, Ait Oumdis, and Ait Tamlil belonged to the Azilal Province (green surface).

LDA results coincidently mirrored the geographical origin of Moroccan saffron samples. Accordingly, Azilal saffron was well discriminated against by Taroudant and Ouarzazate saffron. Moreover, Ouarzazate and Taroudant samples belonged to the same class owing to their geographical closeness, which means they have similar climate and agronomic conditions (as shown in Figure 5). 

**Figure 6:**
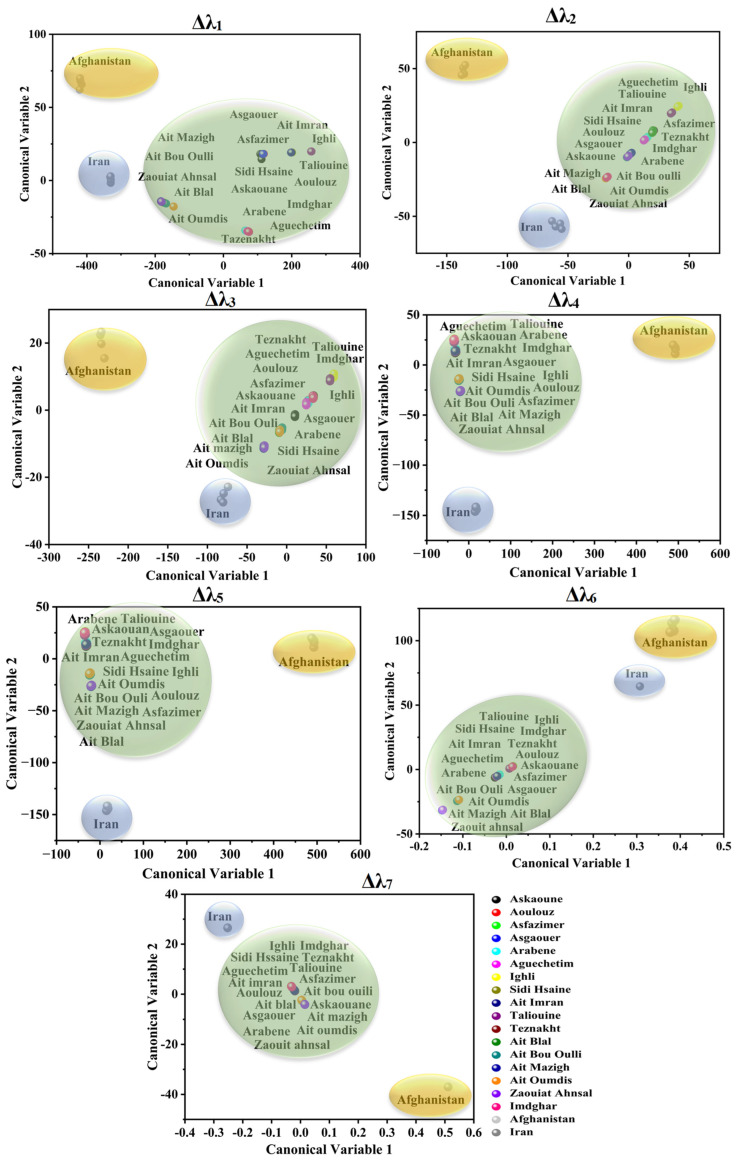
LDA canonical scores applied on Moroccan (green surface), Iranian (blue surface), and Afghan (yellow surface) saffron SFSs using different offsets (Δλ_i_, with *i* = 1 to 7).

Based on Figure 6, the canonical scores of the Afghan, Iranian, and Moroccan saffron samples were correctly assigned according to their geographical location for all the offsets. Thus, each country’s saffron formed a separated class. Significant classification rates ranging from 70% to 100% were achieved by applying LDA, as seen in Table 3. Furthermore, to evaluate the accuracy of the LDA approach, the generated classification models were validated using the cross-validation method by the cancelation of six samples (two samples from each province for Moroccan saffron discrimination and two samples from each country for the international classification). As a result, the classification ability of the LDA models (% of the objects belonging to the testing set correctly classified using the developed model) ranged from 70% to 98%, which revealed that the LDA models showed satisfactory results for the classification of Moroccan saffron samples as well as for the discrimination of Afghan, Iranian, and Moroccan saffron samples. As a whole, these results indicated that LDA can be a robust and accurate method for geographical origin discrimination of saffron samples.

Broadly speaking, PCA and LDA analyses have demonstrated that Afghan saffron was very different from Iranian and Moroccan saffron due to some specific fluorescence ranges (R5 and R6 identified from EEM maps) that were present in Afghan spectra and absent in Moroccan and Iranian saffron spectra (the case of Δλ_4_ and Δλ_5_). These fluorescence ranges generally corresponded to the fluorescence phenomenon of vitamin A, vitamin B2, and terpenoids such as saponins and crocusatins. Moreover, fluorescence ranges (referred to R1 identified by EEM) were present in Moroccan and Iranian saffron spectra but absent in Afghan spectra (the case of Δλ_1_, Δλ_2_, and Δλ_3_), which matched the polyphenolic composition. When considering the common fluorescence ranges, the saffron spectra of Afghan saffron presented higher intensities (the case of Δλ_6_ and Δλ_7_), unlike the UV–vis spectrophotometric measures, which did not differentiate well between Moroccan (Azilal) and Afghan saffron samples. Furthermore, scores of graphical displays illustrated the difference between Iranian and Moroccan saffron, especially samples coming from Taroudant and Ouarzazate provinces. This dissimilarity was due to the low emission intensities measured in Iranian saffron samples. The discrimination obtained between Moroccan, Afghan, and Iranian saffron can be attributed to the manners of saffron cultivation adopted in these countries and also the specific agronomic and climate conditions of each country [7]. Therefore, the proposed methodology successfully distinguished between the reported saffron samples using both picrocrocin, crocin, and safranal content, which was determined by UV–vis spectrophotometry, and fluorophore compounds such as polyphenolic, vitamins, and terpenoids, which were defined by total and synchronous fluorescence.

## 4. Conclusions

The total and synchronous fluorescence modes were performed for the first time for the discrimination of different saffron samples from three provinces in Morocco (Ouarzazate, Taroudante, and Azilal). Moreover, they were employed to differentiate globally between Moroccan, Afghan, and Iranian saffron. Identification of the unique fluorescent fingerprints of each saffron sample was achieved by visualizing EEMs physiognomy. Furthermore, PCA and LDA were demonstrated to be powerful and practical for recognition and grouped the involved SF saffron spectra with remarkable explained variance using only two PCs (97–100%). Moreover, high classification capabilities (70–90%) were demonstrated when applying the LDA method. Certainly, it has been proven that Azilal saffron was well separated from Ouarzazate and Taroudante saffron owing to the high fluorescence intensities registered in the last two provinces. In addition, Afghan, Iranian, and Moroccan saffron samples were significantly separated using the different synchronous offsets, alike, UV–vis spectrophotometry, which did not discriminate well between the investigated samples. 

Considering the merits of the non-destructive fluorescence techniques in this work, including simplicity, sensitivity, rapidity, and reliability, these findings pave the way for the verification of the saffron adulteration and the quantification of fluorophore compounds found in the saffron for its valorization in the cosmetics, food science, and pharmacy fields.

## Figures and Tables

**Table 1:** Moroccan saffron production areas and their geographical coordinates.

Location	Province	Latitude	Longitude	Altitude (m)
Askaoune	Taroudant	30°46′7″ N	7°46′23″ W	1857
Aoulouz		30°42′0″ N	8°9′0″ W	735
Asfazimer		30°42′09″ N	7°45′00″ W	2009
Asgaouer		30°35′53″ N	7°39′8″ W	1829
Arabene		30°49′32″ N	7°45′37″ W	1992
Aguechetim		30°48′27″ N	7°47′18″ W	1930
Ighli		30°34′56″ N	8°33′16″ W	406
Sidi Hsaine		30°28′10″ N	7°46′19″ W	1491
Ait Imran		30°34′35″ N	7°36′34″ W	1687
Taliouine		30°31′58″ N	7°55′32″ W	1029
Imdghar	Ouarzazate	30°60′51″ N	7°36′04″ W	1535
Tazenakht		30°34′25″ N	7°12′10″ W	1410
Ait Mazigh	Azilal	32°4′29″ N	6°21′6″ W	1140
Zaouiat Ahansal		31°49′57″ N	6°06′20″ W	1616
Ait Bou Oulli		31°36′11″ N	6°36′13″ W	1673
Ait Blal		31°41′27″ N	6°42′59″ W	1583
Ait Oumdis		31°29′45″ N	7°03′11″ W	1283
Ait Tamlil		31°28′48″ N	6°56′24″ W	1626

**Table 2:** Calibration/validation explained variance ((var)_c_/(var)_v_) (%) in the principal component analysis applied for different offsets (Δλ_i_, with *i* = 1 to 7).

		Δλ_1_	Δλ_2_	Δλ_3_	Δλ_4_	Δλ_5_	Δλ_6_	Δλ_7_
Moroccan saffron	PC_1_	95/89	97/96	98/97	99/98	97/97	99/98	98/97
	PC_2_	4/7	2/2	0.1/0.2	0.2/1	1/1	0.1/0.5	0.2/0.1
Moroccan, Afghan, and Iranian saffron	PC_1_	88/86	63/60	81/80	71/66	85/80	86/86	94/93
	PC_2_	10/8	34/34	17/5	26/14	14/10	12/12	5/5

**Table 3:** Classification rates (%) in the linear discriminant analysis applied for different offsets (Δλ_i_, with *i* = 1 to 7).

	Δλ_1_	Δλ_2_	Δλ_3_	Δλ_4_	Δλ_5_	Δλ_6_	Δλ_7_
Moroccan saffron	80	99	98	98	95	94	95
Moroccan, Afghan, and Iranian saffron	83	70	83	83	84	95	96

## Data Availability

Not applicable.

## Supplementary Materials

The following supporting information can be downloaded at: https://www.mdpi.com/article/10.3390/foods12091747/s1, Figure S1: UV spectra of Moroccan, Afghan, and Iranian saffron aqueous extracts; Figure S2: EEM maps of Taliouine extracts by assessing four extraction solvents, including H_2_O (100%), Methanol (100%), H_2_O:Methanol (1:1), and H_2_O:Methanol:FA (50:49:1).; Figure S3: EEM maps of Taliouine extracts by assessing four extraction solvents, including H_2_O (100%), Methanol (100%), H_2_O:Methanol (1:1), and H_2_O:Methanol:FA (50:49:1) using 500 mg of saffron and 30 min in the extraction time.; Figure S4: EEMs of Taliouine saffron extracts using different extraction time, they are 5 min, 10 min, 20 min, and 40 min using H_2_O:Methanol:FA as the extraction solvent of 200 mg of saffron; Table S1: Emission-excitation matrix of Moroccan saffron coming from Taroudant, Ouarzazate, and Azilal.
