# Supplementary material for: Geographical Classification of Saffron (Crocus Sativus L.) Using Total and Synchronous Fluorescence Combined with Chemometric Approaches
Source: Foods. 2023 Apr 23;12(9):1747. doi: 10.3390/foods12091747 (PMC10178536; doi:10.3390/foods12091747)
Supplement: Supplementary file 1 [file foods-12-01747-s001.zip › foods-2333572-supplementary.pdf]

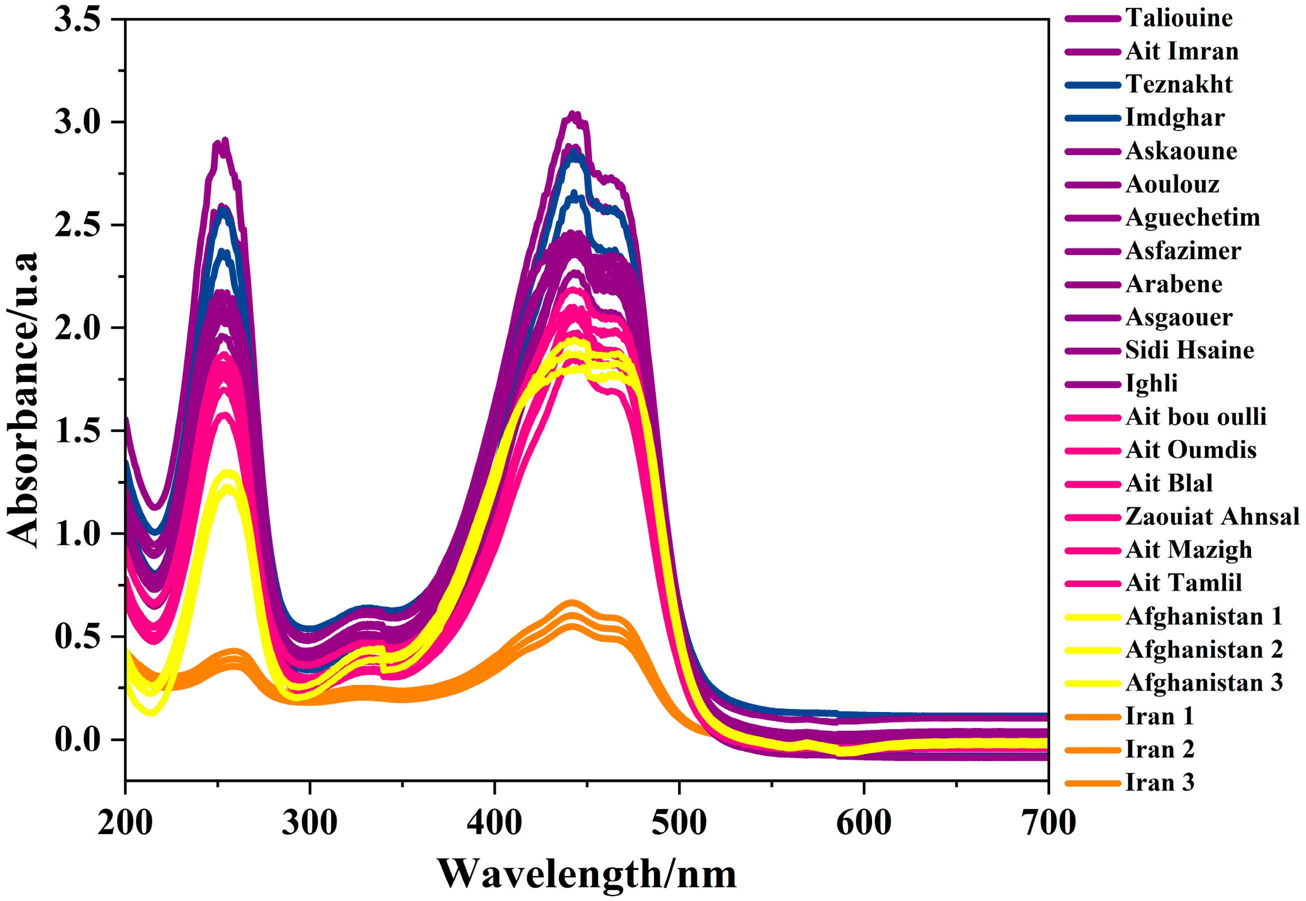


**Figure S1**. UV spectra of Moroccan, Afghan, and Iranian saffron aqueous extracts.

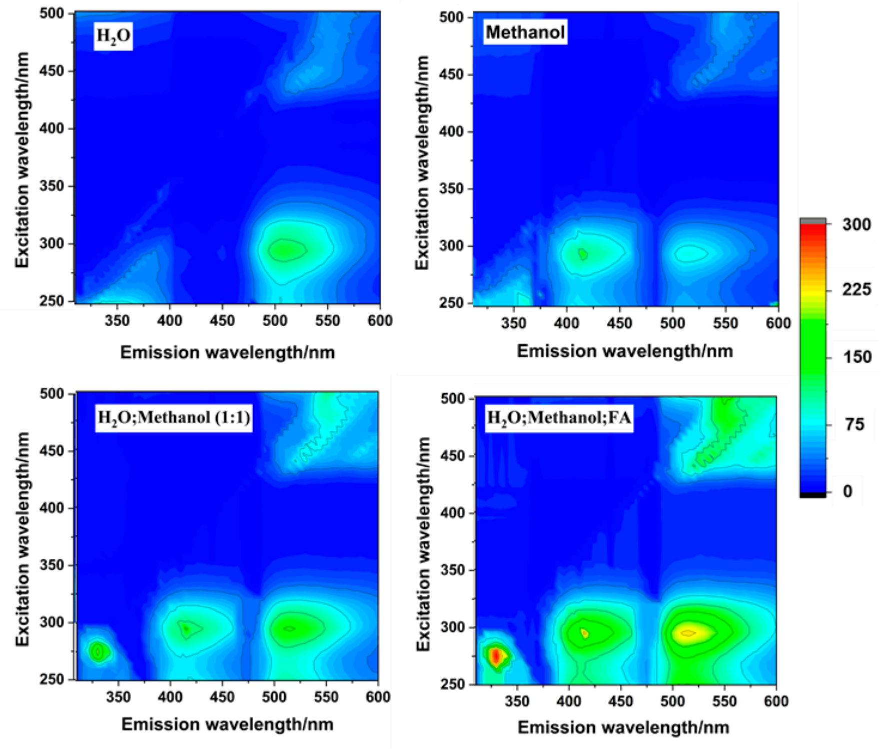


**Figure S2**. EEM maps of Taliouine extracts by assessing four extraction solvents, including $H_2O$ (100%), Methanol (100%), $H_2O$; Methanol (1:1), and $H_2O$; Methanol; FA (50:49:1).

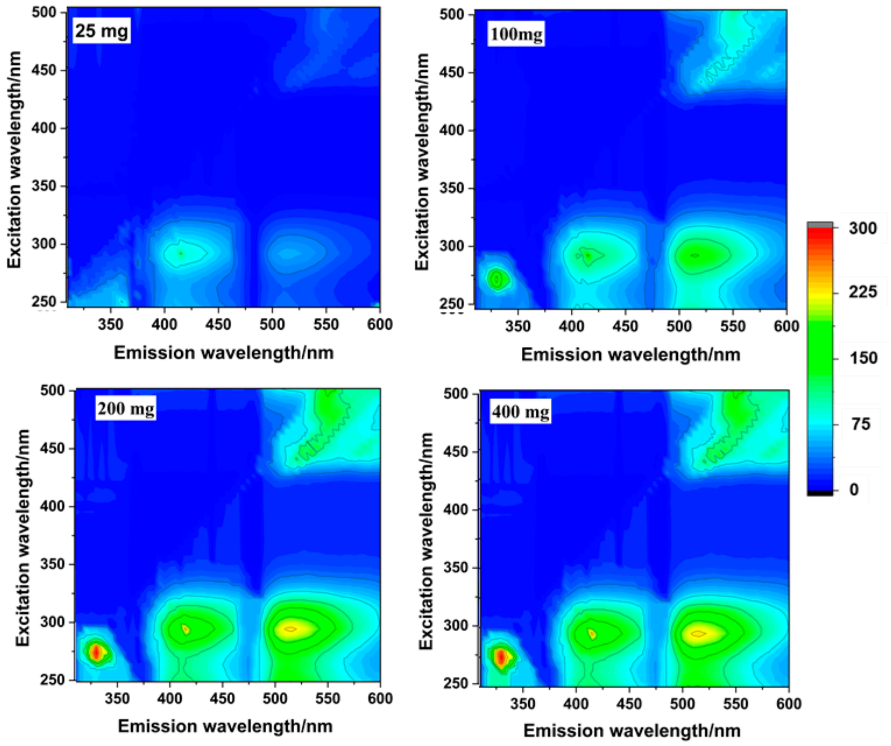

**Figure S3**. EEM maps of Taliouine extracts by assessing four extraction solvents, including $H_2O$ (100%), Methanol (100%), $H_2O$; Methanol (1:1), and $H_2O$; Methanol; FA (50:49:1) using 500 mg of saffron and 30 min in the extraction time.

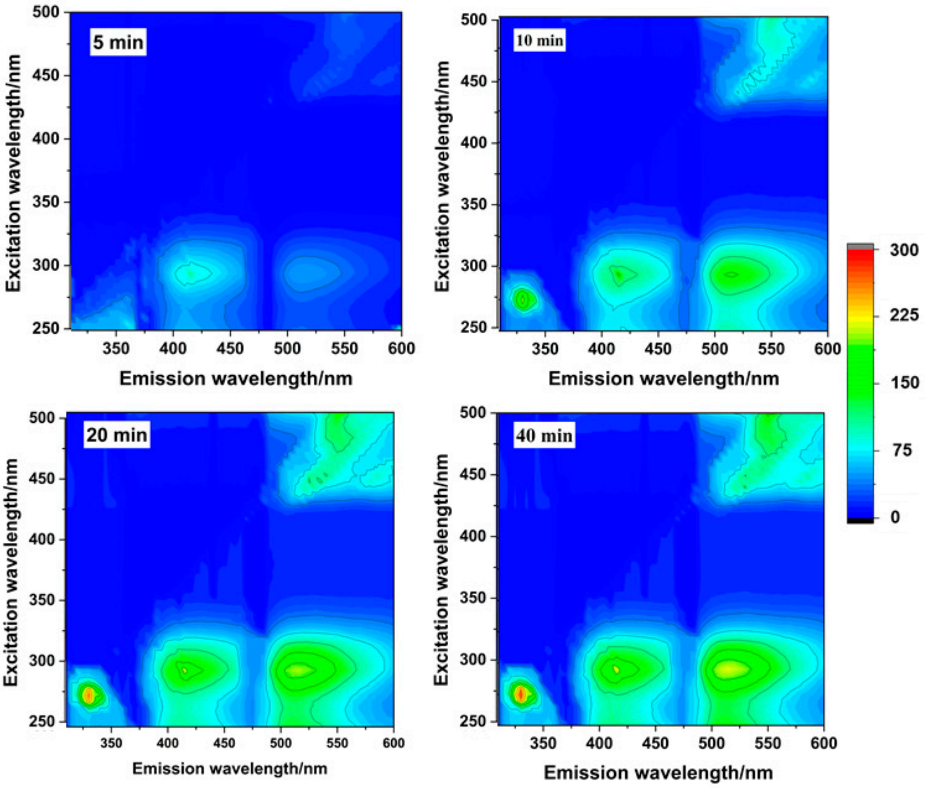


**Figure S4**. EEMs of Taliouine saffron extracts using different extraction time, they are 5 min, 10 min, 20 min, and 40 min using $H_2O$; Methanol; FA as the extraction solvent of 200 mg of saffron.

**Table S1**. Emission-excitation matrix of Moroccan saffron coming from Taroudant, Ouarzazate, and Azilal

| Saffron sample | EEM contours |
|---|---|

| | |
|---|---|
| **Taroudant** | 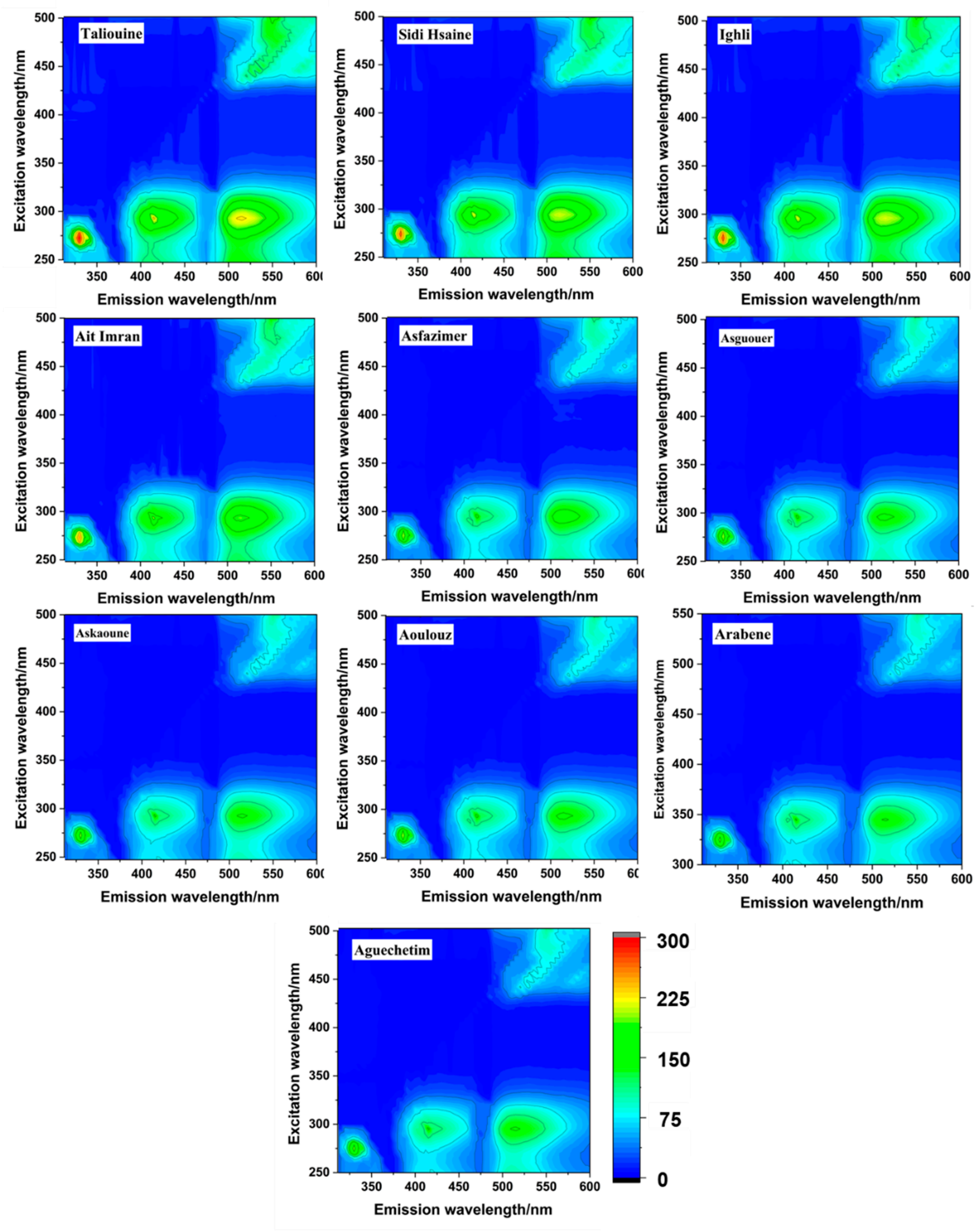 |
| **Ouarzazate** | 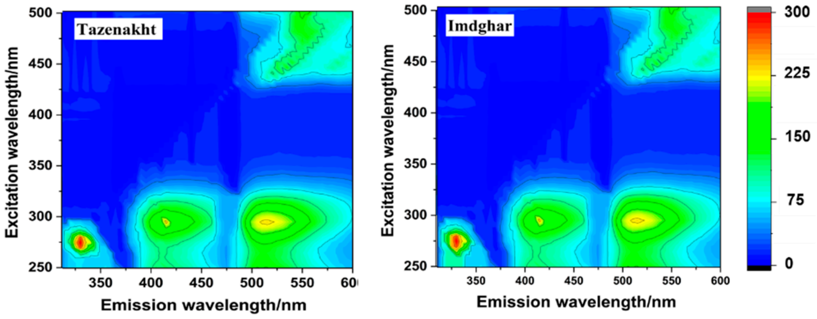 |

**Azilal**

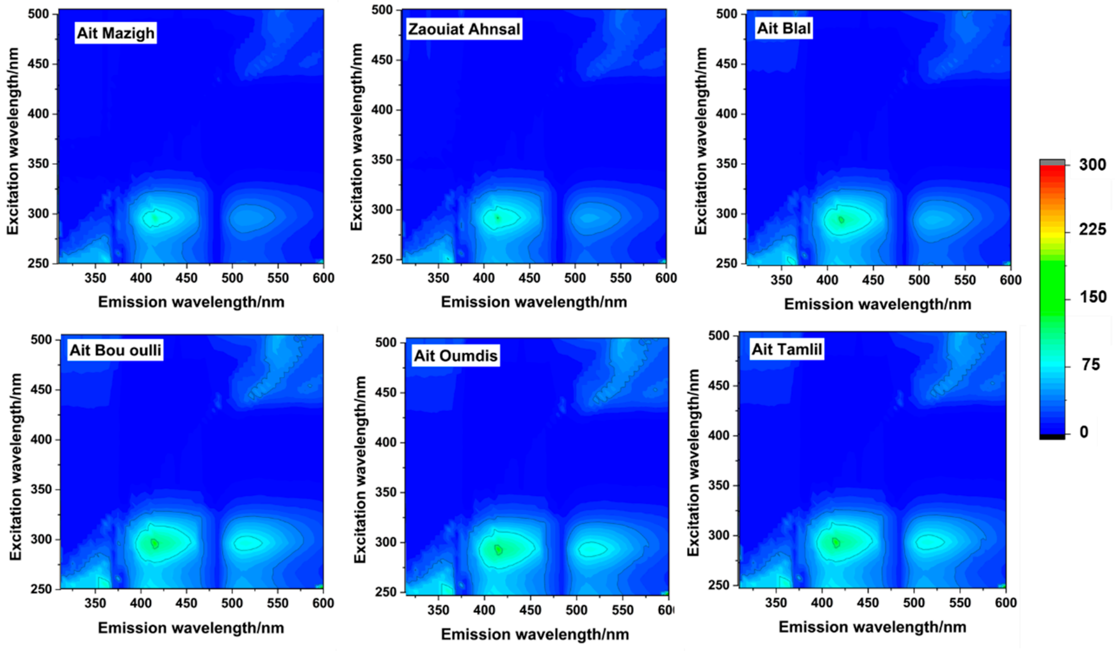

Ait Mazigh
Zaouiat Ahnsal
Ait Blal
Ait Bou oulli
Ait Oumdis
Ait Tamlil
Excitation wavelength/nm
Emission wavelength/nm
300
225
150
75
0
